# (Triphenyl­phosphine-κ*P*)[1,1,1-tris­(diphenyl­phosphinometh­yl)ethane-κ^3^ *P*,*P*′,*P*′′]copper(I) tetra­fluoridoborate

**DOI:** 10.1107/S1600536810007312

**Published:** 2010-03-06

**Authors:** Qi Yin, Xin Gan

**Affiliations:** a College of Chemistry and Chemical Engineering, Yunnan Normal University, Kunming 650092, People’s Republic of China

## Abstract

In the title mononuclear Cu^I^ complex, [Cu(C_18_H_15_P)(C_41_H_39_P_3_)]BF_4_, the cation has a basic rigid core structure reminiscent of the framework of diamond. The metal atom is coordinated by four P atoms in a distorted tetra­hedral geometry, the distortion arising from the steric hindrance of the phenyl groups. The anion is disordered over two positions, with an occupancy ratio of 0.524 (17):0.476 (17). The cations and anions are closely packed in the crystal and are in h.c.p. arrangements.

## Related literature

For the synthesis of related complexes, see: Pawlowski *et al.* (2005 ▶). For the structures of related complexes, see: Kourkine *et al.* (1996 ▶); Mautz *et al.* (2008 ▶).
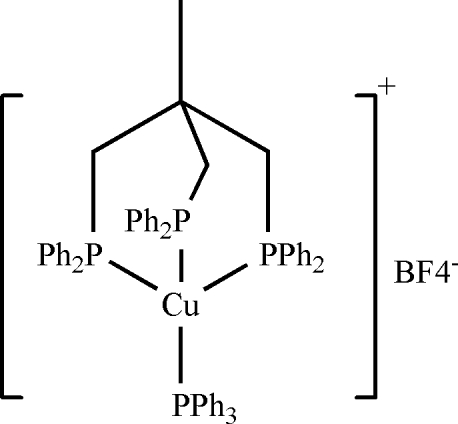


## Experimental

### 

#### Crystal data

[Cu(C_18_H_15_P)(C_41_H_39_P_3_)]BF_4_
*M* *_r_* = 1037.25
Monoclinic, 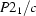

*a* = 13.470 (4) Å
*b* = 14.356 (4) Å
*c* = 26.240 (7) Å
β = 91.338 (5)°
*V* = 5073 (2) Å^3^
*Z* = 4
Mo *K*α radiation
μ = 0.61 mm^−1^
*T* = 293 K
0.20 × 0.16 × 0.14 mm

#### Data collection

Bruker SMART diffractometer
Absorption correction: multi-scan (*SADABS*; Sheldrick, 1996 ▶) *T*_min_ = 0.696, *T*_max_ = 1.000
29232 measured reflections
10469 independent reflections
5826 reflections with *I* > 2σ(*I*)
*R*_int_ = 0.072

#### Refinement

*R*[*F* ^2^ > 2σ(*F* ^2^)] = 0.057
*wR*(*F* ^2^) = 0.138
*S* = 0.98
10469 reflections
660 parameters
H-atom parameters constrained
Δρ_max_ = 0.49 e Å^−3^
Δρ_min_ = −0.31 e Å^−3^

### 

Data collection: *SMART* (Siemens, 1996 ▶); cell refinement: *SAINT* (Siemens, 1996 ▶); data reduction: *SAINT*; program(s) used to solve structure: *SHELXS97* (Sheldrick, 2008 ▶); program(s) used to refine structure: *SHELXL97* (Sheldrick, 2008 ▶); molecular graphics: *ORTEP-3* (Farrugia, 1997 ▶); software used to prepare material for publication: *SHELXTL* (Sheldrick, 2008 ▶).

## Supplementary Material

Crystal structure: contains datablocks I, global. DOI: 10.1107/S1600536810007312/bh2273sup1.cif

Structure factors: contains datablocks I. DOI: 10.1107/S1600536810007312/bh2273Isup2.hkl

Additional supplementary materials:  crystallographic information; 3D view; checkCIF report

## Figures and Tables

**Table:** 

Cu1—P4	2.2852 (11)
Cu1—P3	2.2983 (12)
Cu1—P2	2.3177 (12)
Cu1—P1	2.3314 (12)

**Table:** 

P4—Cu1—P3	122.79 (4)
P4—Cu1—P2	124.27 (4)
P3—Cu1—P2	91.17 (4)
P4—Cu1—P1	117.67 (4)
P3—Cu1—P1	96.58 (4)
P2—Cu1—P1	97.65 (4)

**Fig. 1.**
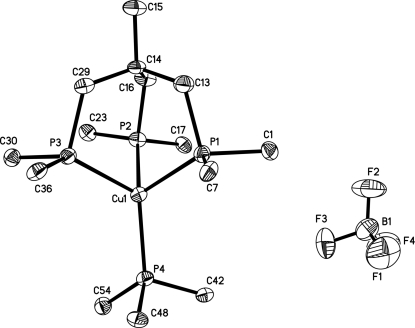
Molecular structure of the title compound. For clarity, all phenyl groups and H atoms are omitted. A single position for the anion is represented.

**Fig. 2.**
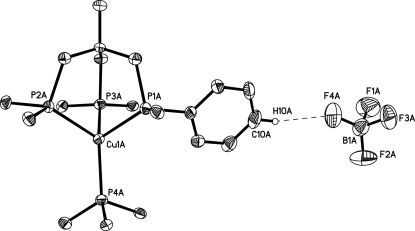
Interactions between cations and anions in the crystal structure of the title compound.

## supplementary crystallographic information

### Comment

As seen in Figure 1, the copper center of the title compound can be described as having a distorted tetrahedron geometry with four of Cu—P bond lengths [2.2852 (11), 2.2983 (12), 2.3177 (12) and 2.3314 (12) Å] and P—Cu—P angles in the range 91.17 (4) to 124.27 (4)°. The average Cu—P distance is thus 2.3082 Å, slightly longer than the corresponding value, 2.2833 Å, reported for a similar compound (Kourkine *et al.*, 1996). The P—Cu—P angles are out of the range 104.0 (1)–116.5 (1)° observed in the same complex.

Tris(diphenylphosphinomethyl)ethane and the Cu^I^ ion compose a bicyclo[2,2,2]octa core with a rigid structure similar to the basic structure of diamond. Interestingly, these rigid cations and disordered anions are connected by C—H···F weak hydrogen bonds, as shown in Figure 2, characterized by a C···F separation of 3.229 (12) Å.

Some other related complexes have been synthesized with Cu^I^ (Pawlowski *et al.*, 2005), and Ni (Mautz *et al.*, 2008).

### Experimental

A mixture of [Cu(CH_3_CN)_4_]BF_4_ (0.1258 g, 0.40 mmol) and 1,1,1-tris(diphenylphosphinomethyl)ethane (triphos, 0.25 g, 0.40 mmol) in dichloromethane (20 mL) was stirred for 3 hours at room temperature under nitrogen atmosphere, and triphenylphosphine (0.1048 g, 0.4 mmol) was then added to the solution. The resulting colorless solution was further stirred for 2 hours and then filtered. The reaction mixture was concentrated in vacuum and the crude product was recrystallized from CH_2_Cl_2_/diethyl ether, to give white crystals (Yield: 82%).

### Refinement

All H atoms were positioned geometrically and refined as riding atoms, with C—H = 0.93 Å and *U*_iso_(H) = 1.2*U*_eq_(C) for aromatic CH, C—H = 0.96 Å and *U*_iso_(H) = 1.5*U*_eq_(C) for the methyl group, and C—H = 0.97 Å and *U*_iso_(H) = 1.2*U*_eq_(C) for the methylene groups. The F atoms in the aion are disordered over two positions, and occupancies were refined with the sum of two disordered sites constrained to unity. The occupation factors converged to 0.476 (17) and 0.524 (17).

### Crystal data

**Table:**

[Cu(C_18_H_15_P)(C_41_H_39_P_3_)]BF_4_	*F*(000) = 2152
*M**_r_* = 1037.25	*D*_x_ = 1.358 Mg m^−^^3^
Monoclinic, *P*2_1_/*c*	Mo *K*α radiation, λ = 0.71073 Å
Hall symbol: -P 2ybc	Cell parameters from 904 reflections
*a* = 13.470 (4) Å	θ = 2.8–23.1°
*b* = 14.356 (4) Å	µ = 0.61 mm^−^^1^
*c* = 26.240 (7) Å	*T* = 293 K
β = 91.338 (5)°	Block, white
*V* = 5073 (2) Å^3^	0.20 × 0.16 × 0.14 mm
*Z* = 4	

### Data collection

**Table:**

Bruker SMART diffractometer	10469 independent reflections
Radiation source: fine-focus sealed tube	5826 reflections with *I* > 2σ(*I*)
graphite	*R*_int_ = 0.072
φ and ω scans	θ_max_ = 26.5°, θ_min_ = 1.6°
Absorption correction: multi-scan (*SADABS*; Sheldrick, 1996)	*h* = −9→16
*T*_min_ = 0.696, *T*_max_ = 1.000	*k* = −17→18
29232 measured reflections	*l* = −29→32

### Refinement

**Table:**

Refinement on *F*^2^	Primary atom site location: structure-invariant direct methods
Least-squares matrix: full	Secondary atom site location: difference Fourier map
*R*[*F*^2^ > 2σ(*F*^2^)] = 0.057	Hydrogen site location: inferred from neighbouring sites
*wR*(*F*^2^) = 0.138	H-atom parameters constrained
*S* = 0.98	*w* = 1/[σ^2^(*F*_o_^2^) + (0.0633*P*)^2^] where *P* = (*F*_o_^2^ + 2*F*_c_^2^)/3
10469 reflections	(Δ/σ)_max_ < 0.001
660 parameters	Δρ_max_ = 0.49 e Å^−^^3^
0 restraints	Δρ_min_ = −0.30 e Å^−^^3^
0 constraints	

### Fractional atomic coordinates and isotropic or equivalent isotropic displacement parameters (Å^2^)

**Table:**

	*x*	*y*	*z*	*U*_iso_*/*U*_eq_	Occ. (<1)
Cu1	0.24249 (3)	0.75782 (3)	0.396487 (17)	0.03077 (13)	
P1	0.41107 (7)	0.73412 (7)	0.41402 (4)	0.0339 (2)	
P2	0.25203 (7)	0.77281 (7)	0.30879 (4)	0.0335 (2)	
P3	0.24433 (7)	0.91686 (6)	0.40649 (4)	0.0324 (2)	
P4	0.13205 (7)	0.66350 (7)	0.43651 (4)	0.0338 (2)	
C1	0.4799 (3)	0.6276 (3)	0.39782 (16)	0.0401 (10)	
C2	0.4919 (4)	0.6035 (3)	0.34792 (18)	0.0666 (14)	
H2	0.4660	0.6419	0.3223	0.080*	
C3	0.5416 (4)	0.5238 (4)	0.3349 (2)	0.0859 (18)	
H3	0.5493	0.5088	0.3007	0.103*	
C4	0.5797 (4)	0.4666 (4)	0.3719 (2)	0.0820 (17)	
H4	0.6125	0.4120	0.3631	0.098*	
C5	0.5693 (4)	0.4898 (3)	0.4216 (2)	0.0736 (15)	
H5	0.5960	0.4513	0.4469	0.088*	
C6	0.5202 (3)	0.5692 (3)	0.43494 (18)	0.0558 (12)	
H6	0.5139	0.5841	0.4692	0.067*	
C7	0.4547 (3)	0.7550 (2)	0.47942 (15)	0.0388 (9)	
C8	0.5539 (3)	0.7766 (3)	0.49112 (18)	0.0499 (11)	
H8	0.5999	0.7793	0.4653	0.060*	
C9	0.5836 (4)	0.7940 (3)	0.5406 (2)	0.0663 (15)	
H9	0.6492	0.8105	0.5477	0.080*	
C10	0.5184 (4)	0.7875 (3)	0.5799 (2)	0.0691 (15)	
H10	0.5399	0.7977	0.6134	0.083*	
C11	0.4214 (4)	0.7657 (3)	0.56900 (18)	0.0618 (13)	
H11	0.3762	0.7621	0.5952	0.074*	
C12	0.3901 (3)	0.7490 (3)	0.51913 (16)	0.0464 (10)	
H12	0.3241	0.7334	0.5124	0.056*	
C13	0.4777 (3)	0.8240 (3)	0.37731 (16)	0.0436 (10)	
H13A	0.5145	0.8623	0.4017	0.052*	
H13B	0.5263	0.7920	0.3569	0.052*	
C14	0.4194 (2)	0.8901 (2)	0.34159 (14)	0.0325 (9)	
C15	0.4987 (3)	0.9517 (3)	0.31621 (16)	0.0482 (11)	
H15A	0.4664	0.9974	0.2949	0.072*	
H15B	0.5407	0.9134	0.2959	0.072*	
H15C	0.5381	0.9824	0.3421	0.072*	
C16	0.3684 (3)	0.8362 (3)	0.29736 (15)	0.0440 (10)	
H16A	0.4160	0.7916	0.2848	0.053*	
H16B	0.3551	0.8803	0.2700	0.053*	
C17	0.2630 (3)	0.6659 (3)	0.27227 (15)	0.0376 (9)	
C18	0.2852 (3)	0.6658 (3)	0.22080 (16)	0.0487 (11)	
H18	0.2976	0.7217	0.2042	0.058*	
C19	0.2888 (3)	0.5840 (4)	0.19470 (18)	0.0608 (13)	
H19	0.3024	0.5847	0.1601	0.073*	
C20	0.2729 (3)	0.5008 (3)	0.2185 (2)	0.0582 (13)	
H20	0.2751	0.4455	0.2001	0.070*	
C21	0.2537 (3)	0.4988 (3)	0.2695 (2)	0.0587 (13)	
H21	0.2443	0.4423	0.2860	0.070*	
C22	0.2483 (3)	0.5820 (3)	0.29635 (17)	0.0496 (11)	
H22	0.2347	0.5810	0.3309	0.060*	
C23	0.1601 (3)	0.8362 (3)	0.26973 (14)	0.0379 (9)	
C24	0.0716 (3)	0.7921 (3)	0.25629 (15)	0.0472 (11)	
H24	0.0624	0.7298	0.2648	0.057*	
C25	−0.0038 (3)	0.8402 (4)	0.23009 (17)	0.0635 (14)	
H25	−0.0639	0.8112	0.2222	0.076*	
C26	0.0124 (4)	0.9319 (4)	0.21603 (18)	0.0716 (16)	
H26	−0.0373	0.9643	0.1983	0.086*	
C27	0.0991 (4)	0.9750 (4)	0.22772 (19)	0.0705 (15)	
H27	0.1093	1.0362	0.2174	0.085*	
C28	0.1733 (3)	0.9280 (3)	0.25509 (16)	0.0513 (11)	
H28	0.2322	0.9585	0.2636	0.062*	
C29	0.3536 (3)	0.9585 (3)	0.37104 (15)	0.0409 (10)	
H29A	0.3296	1.0048	0.3468	0.049*	
H29B	0.3966	0.9909	0.3953	0.049*	
C30	0.1404 (3)	0.9788 (3)	0.37651 (14)	0.0362 (9)	
C31	0.1473 (3)	1.0711 (3)	0.36160 (17)	0.0522 (11)	
H31	0.2063	1.1036	0.3672	0.063*	
C32	0.0671 (4)	1.1148 (3)	0.33846 (19)	0.0656 (14)	
H32	0.0725	1.1765	0.3280	0.079*	
C33	−0.0198 (4)	1.0681 (4)	0.33079 (18)	0.0623 (13)	
H33	−0.0734	1.0981	0.3149	0.075*	
C34	−0.0292 (3)	0.9778 (3)	0.34612 (16)	0.0515 (11)	
H34	−0.0892	0.9466	0.3411	0.062*	
C35	0.0507 (3)	0.9329 (3)	0.36909 (15)	0.0424 (10)	
H35	0.0443	0.8714	0.3797	0.051*	
C36	0.2550 (3)	0.9773 (2)	0.46789 (15)	0.0389 (9)	
C37	0.1756 (3)	1.0240 (3)	0.48823 (17)	0.0597 (13)	
H37	0.1167	1.0297	0.4693	0.072*	
C38	0.1828 (4)	1.0628 (4)	0.5367 (2)	0.0768 (16)	
H38	0.1291	1.0950	0.5497	0.092*	
C39	0.2678 (5)	1.0540 (4)	0.56521 (19)	0.0737 (16)	
H39	0.2722	1.0800	0.5976	0.088*	
C40	0.3463 (4)	1.0072 (3)	0.54629 (19)	0.0656 (14)	
H40	0.4042	1.0005	0.5659	0.079*	
C41	0.3402 (3)	0.9696 (3)	0.49786 (17)	0.0512 (11)	
H41	0.3948	0.9383	0.4852	0.061*	
C42	0.1589 (3)	0.5405 (2)	0.42803 (14)	0.0347 (9)	
C43	0.2571 (3)	0.5113 (3)	0.43182 (19)	0.0577 (13)	
H43	0.3061	0.5539	0.4414	0.069*	
C44	0.2832 (4)	0.4211 (3)	0.4217 (2)	0.0725 (16)	
H44	0.3493	0.4028	0.4245	0.087*	
C45	0.2116 (4)	0.3578 (3)	0.40737 (19)	0.0639 (13)	
H45	0.2291	0.2968	0.3997	0.077*	
C46	0.1148 (4)	0.3845 (3)	0.40442 (18)	0.0575 (12)	
H46	0.0662	0.3410	0.3957	0.069*	
C47	0.0879 (3)	0.4755 (3)	0.41422 (16)	0.0455 (10)	
H47	0.0215	0.4929	0.4115	0.055*	
C48	0.1349 (3)	0.6788 (3)	0.50546 (15)	0.0387 (10)	
C49	0.1545 (3)	0.6084 (3)	0.53999 (16)	0.0501 (11)	
H49	0.1645	0.5479	0.5285	0.060*	
C50	0.1593 (3)	0.6276 (4)	0.59191 (18)	0.0688 (15)	
H50	0.1718	0.5795	0.6149	0.083*	
C51	0.1462 (4)	0.7142 (5)	0.6093 (2)	0.0836 (19)	
H51	0.1499	0.7258	0.6442	0.100*	
C52	0.1273 (4)	0.7863 (4)	0.5757 (2)	0.0791 (17)	
H52	0.1181	0.8465	0.5878	0.095*	
C53	0.1219 (3)	0.7687 (3)	0.52404 (18)	0.0581 (12)	
H53	0.1096	0.8174	0.5014	0.070*	
C54	−0.0001 (3)	0.6724 (3)	0.41974 (16)	0.0397 (10)	
C55	−0.0735 (3)	0.6595 (3)	0.45486 (18)	0.0572 (12)	
H55	−0.0565	0.6485	0.4889	0.069*	
C56	−0.1728 (4)	0.6631 (4)	0.4393 (2)	0.0783 (16)	
H56	−0.2220	0.6532	0.4630	0.094*	
C57	−0.1987 (4)	0.6811 (3)	0.3895 (3)	0.0739 (16)	
H57	−0.2653	0.6836	0.3795	0.089*	
C58	−0.1262 (4)	0.6953 (3)	0.3544 (2)	0.0700 (14)	
H58	−0.1432	0.7081	0.3206	0.084*	
C59	−0.0277 (3)	0.6904 (3)	0.36998 (18)	0.0561 (12)	
H59	0.0214	0.6995	0.3461	0.067*	
B1	0.3312 (7)	0.2119 (6)	0.2671 (3)	0.080 (2)	
F1	0.299 (2)	0.128 (2)	0.2471 (10)	0.143 (9)	0.476 (17)
F2	0.3933 (14)	0.2505 (10)	0.2382 (7)	0.152 (7)	0.476 (17)
F3	0.2403 (7)	0.2555 (7)	0.2643 (6)	0.120 (5)	0.476 (17)
F4	0.3505 (12)	0.1988 (13)	0.3157 (5)	0.160 (8)	0.476 (17)
F1'	0.326 (2)	0.1207 (16)	0.2673 (9)	0.135 (9)	0.524 (17)
F2'	0.4334 (11)	0.2230 (11)	0.2776 (7)	0.196 (7)	0.524 (17)
F3'	0.3244 (12)	0.2558 (9)	0.2234 (5)	0.140 (6)	0.524 (17)
F4'	0.2890 (13)	0.2538 (8)	0.3048 (6)	0.164 (8)	0.524 (17)

### Atomic displacement parameters (Å^2^)

**Table:**

	*U*^11^	*U*^22^	*U*^33^	*U*^12^	*U*^13^	*U*^23^
Cu1	0.0327 (3)	0.0303 (3)	0.0293 (2)	−0.00246 (19)	−0.00001 (17)	0.0021 (2)
P1	0.0303 (5)	0.0334 (5)	0.0378 (6)	0.0010 (4)	−0.0031 (4)	0.0056 (4)
P2	0.0333 (6)	0.0401 (6)	0.0271 (5)	−0.0041 (4)	−0.0015 (4)	0.0001 (4)
P3	0.0351 (6)	0.0281 (5)	0.0342 (6)	−0.0019 (4)	0.0021 (4)	0.0008 (4)
P4	0.0363 (6)	0.0314 (5)	0.0339 (6)	−0.0049 (4)	0.0025 (4)	0.0015 (4)
C1	0.030 (2)	0.041 (2)	0.049 (3)	0.0023 (17)	−0.0009 (18)	0.000 (2)
C2	0.085 (4)	0.065 (3)	0.050 (3)	0.033 (3)	−0.009 (3)	−0.002 (3)
C3	0.114 (5)	0.087 (4)	0.057 (4)	0.046 (4)	−0.001 (3)	−0.015 (3)
C4	0.099 (4)	0.065 (4)	0.083 (5)	0.043 (3)	0.015 (3)	−0.003 (3)
C5	0.088 (4)	0.059 (3)	0.074 (4)	0.032 (3)	0.005 (3)	0.021 (3)
C6	0.068 (3)	0.044 (3)	0.055 (3)	0.010 (2)	0.005 (2)	0.008 (2)
C7	0.042 (2)	0.030 (2)	0.044 (2)	−0.0005 (18)	−0.0092 (18)	0.0071 (18)
C8	0.043 (3)	0.050 (3)	0.056 (3)	−0.004 (2)	−0.010 (2)	0.009 (2)
C9	0.071 (4)	0.051 (3)	0.075 (4)	−0.009 (2)	−0.041 (3)	0.011 (3)
C10	0.106 (5)	0.051 (3)	0.049 (3)	−0.004 (3)	−0.029 (3)	0.000 (2)
C11	0.085 (4)	0.056 (3)	0.044 (3)	−0.003 (3)	0.000 (3)	0.001 (2)
C12	0.051 (3)	0.040 (2)	0.049 (3)	−0.0064 (19)	−0.002 (2)	0.004 (2)
C13	0.035 (2)	0.050 (3)	0.046 (3)	−0.0034 (18)	0.0001 (18)	0.012 (2)
C14	0.027 (2)	0.035 (2)	0.035 (2)	−0.0089 (16)	0.0017 (16)	0.0066 (17)
C15	0.039 (2)	0.059 (3)	0.047 (3)	−0.015 (2)	0.0043 (19)	0.011 (2)
C16	0.037 (2)	0.061 (3)	0.035 (2)	−0.011 (2)	0.0022 (17)	0.005 (2)
C17	0.031 (2)	0.045 (2)	0.037 (2)	−0.0004 (17)	0.0005 (17)	−0.0076 (19)
C18	0.058 (3)	0.050 (3)	0.038 (3)	0.013 (2)	0.004 (2)	−0.003 (2)
C19	0.061 (3)	0.079 (4)	0.041 (3)	0.023 (3)	−0.001 (2)	−0.013 (3)
C20	0.040 (3)	0.062 (3)	0.072 (4)	0.013 (2)	−0.002 (2)	−0.028 (3)
C21	0.057 (3)	0.043 (3)	0.077 (4)	−0.007 (2)	0.007 (3)	−0.003 (3)
C22	0.058 (3)	0.049 (3)	0.042 (3)	−0.004 (2)	0.007 (2)	−0.002 (2)
C23	0.041 (2)	0.049 (2)	0.024 (2)	0.0014 (19)	0.0006 (16)	−0.0043 (18)
C24	0.046 (3)	0.058 (3)	0.037 (3)	0.008 (2)	0.000 (2)	−0.014 (2)
C25	0.050 (3)	0.092 (4)	0.048 (3)	0.018 (3)	−0.014 (2)	−0.027 (3)
C26	0.082 (4)	0.087 (4)	0.044 (3)	0.043 (3)	−0.013 (3)	−0.004 (3)
C27	0.091 (4)	0.071 (4)	0.050 (3)	0.024 (3)	0.006 (3)	0.010 (3)
C28	0.062 (3)	0.052 (3)	0.041 (3)	0.006 (2)	0.001 (2)	0.005 (2)
C29	0.041 (2)	0.037 (2)	0.045 (3)	−0.0085 (18)	0.0060 (18)	0.0058 (19)
C30	0.041 (2)	0.034 (2)	0.034 (2)	0.0019 (18)	0.0012 (17)	−0.0016 (17)
C31	0.054 (3)	0.039 (2)	0.064 (3)	0.004 (2)	−0.004 (2)	−0.001 (2)
C32	0.077 (4)	0.042 (3)	0.078 (4)	0.020 (3)	−0.003 (3)	0.010 (2)
C33	0.056 (3)	0.071 (4)	0.060 (3)	0.027 (3)	−0.002 (2)	0.001 (3)
C34	0.037 (3)	0.070 (3)	0.048 (3)	0.009 (2)	0.003 (2)	−0.004 (2)
C35	0.046 (3)	0.041 (2)	0.041 (3)	0.0019 (19)	0.0066 (19)	−0.0001 (19)
C36	0.049 (3)	0.028 (2)	0.039 (2)	−0.0092 (18)	0.0011 (19)	0.0021 (17)
C37	0.060 (3)	0.075 (3)	0.044 (3)	0.000 (2)	0.008 (2)	−0.010 (2)
C38	0.085 (4)	0.092 (4)	0.054 (4)	−0.001 (3)	0.020 (3)	−0.018 (3)
C39	0.120 (5)	0.061 (3)	0.041 (3)	−0.024 (3)	−0.001 (3)	−0.009 (3)
C40	0.090 (4)	0.047 (3)	0.058 (3)	−0.013 (3)	−0.027 (3)	0.001 (2)
C41	0.062 (3)	0.035 (2)	0.056 (3)	−0.007 (2)	−0.009 (2)	−0.003 (2)
C42	0.037 (2)	0.037 (2)	0.031 (2)	−0.0050 (17)	0.0021 (16)	0.0057 (17)
C43	0.045 (3)	0.039 (3)	0.090 (4)	−0.004 (2)	−0.004 (2)	0.011 (2)
C44	0.050 (3)	0.053 (3)	0.115 (5)	0.011 (2)	0.007 (3)	0.012 (3)
C45	0.081 (4)	0.040 (3)	0.071 (4)	0.012 (3)	−0.003 (3)	−0.002 (2)
C46	0.067 (3)	0.038 (3)	0.067 (3)	−0.004 (2)	−0.013 (2)	−0.008 (2)
C47	0.042 (3)	0.040 (2)	0.054 (3)	−0.0003 (19)	−0.004 (2)	−0.001 (2)
C48	0.034 (2)	0.045 (2)	0.038 (2)	−0.0110 (18)	0.0053 (17)	−0.0031 (19)
C49	0.047 (3)	0.065 (3)	0.039 (3)	−0.012 (2)	−0.0017 (19)	0.008 (2)
C50	0.055 (3)	0.116 (5)	0.035 (3)	−0.018 (3)	−0.005 (2)	0.013 (3)
C51	0.069 (4)	0.141 (6)	0.041 (3)	−0.041 (4)	0.007 (3)	−0.024 (4)
C52	0.086 (4)	0.090 (4)	0.063 (4)	−0.024 (3)	0.025 (3)	−0.037 (3)
C53	0.070 (3)	0.052 (3)	0.053 (3)	−0.011 (2)	0.014 (2)	−0.004 (2)
C54	0.039 (2)	0.034 (2)	0.046 (3)	−0.0029 (17)	−0.0001 (19)	−0.0031 (19)
C55	0.044 (3)	0.070 (3)	0.057 (3)	0.006 (2)	0.007 (2)	0.006 (2)
C56	0.042 (3)	0.094 (4)	0.099 (5)	0.004 (3)	0.012 (3)	0.008 (4)
C57	0.044 (3)	0.070 (4)	0.107 (5)	0.009 (2)	−0.019 (3)	−0.004 (3)
C58	0.063 (4)	0.079 (4)	0.067 (4)	−0.001 (3)	−0.028 (3)	0.000 (3)
C59	0.052 (3)	0.067 (3)	0.049 (3)	−0.009 (2)	−0.005 (2)	0.001 (2)
B1	0.099 (7)	0.076 (5)	0.066 (5)	−0.006 (5)	−0.007 (5)	0.004 (4)
F1	0.178 (14)	0.120 (17)	0.131 (17)	−0.055 (12)	−0.007 (11)	−0.015 (12)
F2	0.137 (14)	0.164 (11)	0.157 (19)	−0.049 (12)	0.063 (12)	0.030 (12)
F3	0.092 (7)	0.122 (7)	0.146 (13)	0.041 (5)	−0.023 (7)	0.006 (7)
F4	0.152 (13)	0.211 (17)	0.114 (11)	−0.023 (10)	−0.060 (9)	0.032 (10)
F1'	0.192 (19)	0.076 (9)	0.141 (19)	0.017 (11)	0.048 (14)	0.028 (11)
F2'	0.151 (12)	0.254 (15)	0.182 (15)	−0.042 (9)	−0.029 (11)	0.041 (12)
F3'	0.164 (15)	0.127 (7)	0.127 (9)	−0.027 (9)	−0.035 (11)	0.040 (6)
F4'	0.208 (18)	0.145 (10)	0.141 (14)	0.025 (9)	0.063 (14)	−0.041 (10)

### Geometric parameters (Å, °)

**Table:**

Cu1—P4	2.2852 (11)	C27—C28	1.391 (6)
Cu1—P3	2.2983 (12)	C27—H27	0.9300
Cu1—P2	2.3177 (12)	C28—H28	0.9300
Cu1—P1	2.3314 (12)	C29—H29A	0.9700
P1—C7	1.826 (4)	C29—H29B	0.9700
P1—C1	1.843 (4)	C30—C31	1.386 (5)
P1—C13	1.855 (4)	C30—C35	1.386 (5)
P2—C17	1.818 (4)	C31—C32	1.378 (6)
P2—C23	1.831 (4)	C31—H31	0.9300
P2—C16	1.843 (4)	C32—C33	1.360 (6)
P3—C30	1.821 (4)	C32—H32	0.9300
P3—C36	1.833 (4)	C33—C34	1.364 (6)
P3—C29	1.859 (3)	C33—H33	0.9300
P4—C42	1.817 (4)	C34—C35	1.381 (5)
P4—C48	1.822 (4)	C34—H34	0.9300
P4—C54	1.828 (4)	C35—H35	0.9300
C1—C2	1.367 (6)	C36—C41	1.380 (5)
C1—C6	1.387 (6)	C36—C37	1.381 (5)
C2—C3	1.374 (6)	C37—C38	1.389 (6)
C2—H2	0.9300	C37—H37	0.9300
C3—C4	1.363 (7)	C38—C39	1.359 (7)
C3—H3	0.9300	C38—H38	0.9300
C4—C5	1.356 (7)	C39—C40	1.356 (7)
C4—H4	0.9300	C39—H39	0.9300
C5—C6	1.367 (6)	C40—C41	1.382 (6)
C5—H5	0.9300	C40—H40	0.9300
C6—H6	0.9300	C41—H41	0.9300
C7—C12	1.376 (5)	C42—C47	1.379 (5)
C7—C8	1.399 (5)	C42—C43	1.388 (5)
C8—C9	1.373 (6)	C43—C44	1.371 (6)
C8—H8	0.9300	C43—H43	0.9300
C9—C10	1.372 (7)	C44—C45	1.371 (6)
C9—H9	0.9300	C44—H44	0.9300
C10—C11	1.368 (7)	C45—C46	1.358 (6)
C10—H10	0.9300	C45—H45	0.9300
C11—C12	1.386 (6)	C46—C47	1.381 (5)
C11—H11	0.9300	C46—H46	0.9300
C12—H12	0.9300	C47—H47	0.9300
C13—C14	1.537 (5)	C48—C49	1.378 (5)
C13—H13A	0.9700	C48—C53	1.393 (5)
C13—H13B	0.9700	C49—C50	1.390 (6)
C14—C29	1.542 (5)	C49—H49	0.9300
C14—C16	1.543 (5)	C50—C51	1.337 (7)
C14—C15	1.549 (5)	C50—H50	0.9300
C15—H15A	0.9600	C51—C52	1.380 (8)
C15—H15B	0.9600	C51—H51	0.9300
C15—H15C	0.9600	C52—C53	1.378 (7)
C16—H16A	0.9700	C52—H52	0.9300
C16—H16B	0.9700	C53—H53	0.9300
C17—C22	1.376 (5)	C54—C59	1.373 (6)
C17—C18	1.390 (5)	C54—C55	1.380 (5)
C18—C19	1.362 (6)	C55—C56	1.390 (6)
C18—H18	0.9300	C55—H55	0.9300
C19—C20	1.368 (6)	C56—C57	1.370 (7)
C19—H19	0.9300	C56—H56	0.9300
C20—C21	1.367 (6)	C57—C58	1.374 (7)
C20—H20	0.9300	C57—H57	0.9300
C21—C22	1.390 (6)	C58—C59	1.381 (6)
C21—H21	0.9300	C58—H58	0.9300
C22—H22	0.9300	C59—H59	0.9300
C23—C28	1.385 (5)	B1—F1	1.38 (3)
C23—C24	1.389 (5)	B1—F2	1.271 (14)
C24—C25	1.396 (6)	B1—F3	1.375 (12)
C24—H24	0.9300	B1—F4	1.309 (14)
C25—C26	1.386 (7)	B1—F1'	1.31 (2)
C25—H25	0.9300	B1—F2'	1.407 (14)
C26—C27	1.351 (7)	B1—F3'	1.312 (14)
C26—H26	0.9300	B1—F4'	1.299 (12)
			
P4—Cu1—P3	122.79 (4)	C27—C26—C25	121.0 (5)
P4—Cu1—P2	124.27 (4)	C27—C26—H26	119.5
P3—Cu1—P2	91.17 (4)	C25—C26—H26	119.5
P4—Cu1—P1	117.67 (4)	C26—C27—C28	120.1 (5)
P3—Cu1—P1	96.58 (4)	C26—C27—H27	119.9
P2—Cu1—P1	97.65 (4)	C28—C27—H27	119.9
C7—P1—C1	101.56 (18)	C23—C28—C27	120.6 (5)
C7—P1—C13	103.00 (18)	C23—C28—H28	119.7
C1—P1—C13	101.86 (18)	C27—C28—H28	119.7
C7—P1—Cu1	116.88 (13)	C14—C29—P3	121.1 (2)
C1—P1—Cu1	124.63 (13)	C14—C29—H29A	107.0
C13—P1—Cu1	106.09 (12)	P3—C29—H29A	107.0
C17—P2—C23	100.77 (17)	C14—C29—H29B	107.0
C17—P2—C16	104.58 (18)	P3—C29—H29B	107.0
C23—P2—C16	103.28 (18)	H29A—C29—H29B	106.8
C17—P2—Cu1	116.87 (13)	C31—C30—C35	118.6 (4)
C23—P2—Cu1	123.33 (12)	C31—C30—P3	122.2 (3)
C16—P2—Cu1	105.93 (13)	C35—C30—P3	119.2 (3)
C30—P3—C36	101.10 (18)	C32—C31—C30	120.2 (4)
C30—P3—C29	103.67 (18)	C32—C31—H31	119.9
C36—P3—C29	103.94 (18)	C30—C31—H31	119.9
C30—P3—Cu1	115.43 (13)	C33—C32—C31	120.3 (4)
C36—P3—Cu1	124.83 (12)	C33—C32—H32	119.9
C29—P3—Cu1	105.61 (12)	C31—C32—H32	119.9
C42—P4—C48	103.82 (17)	C32—C33—C34	120.6 (4)
C42—P4—C54	103.55 (17)	C32—C33—H33	119.7
C48—P4—C54	103.19 (18)	C34—C33—H33	119.7
C42—P4—Cu1	112.70 (12)	C33—C34—C35	119.7 (4)
C48—P4—Cu1	112.73 (12)	C33—C34—H34	120.1
C54—P4—Cu1	119.24 (13)	C35—C34—H34	120.1
C2—C1—C6	117.8 (4)	C34—C35—C30	120.5 (4)
C2—C1—P1	120.1 (3)	C34—C35—H35	119.7
C6—C1—P1	122.1 (3)	C30—C35—H35	119.7
C1—C2—C3	121.2 (5)	C41—C36—C37	117.4 (4)
C1—C2—H2	119.4	C41—C36—P3	120.7 (3)
C3—C2—H2	119.4	C37—C36—P3	121.6 (3)
C4—C3—C2	120.1 (5)	C36—C37—C38	120.7 (5)
C4—C3—H3	119.9	C36—C37—H37	119.7
C2—C3—H3	119.9	C38—C37—H37	119.7
C5—C4—C3	119.4 (5)	C39—C38—C37	120.5 (5)
C5—C4—H4	120.3	C39—C38—H38	119.8
C3—C4—H4	120.3	C37—C38—H38	119.8
C4—C5—C6	120.9 (5)	C40—C39—C38	119.9 (5)
C4—C5—H5	119.5	C40—C39—H39	120.1
C6—C5—H5	119.5	C38—C39—H39	120.1
C5—C6—C1	120.5 (5)	C39—C40—C41	120.0 (5)
C5—C6—H6	119.7	C39—C40—H40	120.0
C1—C6—H6	119.7	C41—C40—H40	120.0
C12—C7—C8	117.7 (4)	C36—C41—C40	121.6 (4)
C12—C7—P1	120.4 (3)	C36—C41—H41	119.2
C8—C7—P1	121.8 (3)	C40—C41—H41	119.2
C9—C8—C7	120.2 (4)	C47—C42—C43	117.9 (4)
C9—C8—H8	119.9	C47—C42—P4	123.5 (3)
C7—C8—H8	119.9	C43—C42—P4	118.5 (3)
C10—C9—C8	121.4 (5)	C44—C43—C42	121.3 (4)
C10—C9—H9	119.3	C44—C43—H43	119.3
C8—C9—H9	119.3	C42—C43—H43	119.3
C11—C10—C9	118.9 (5)	C43—C44—C45	119.8 (4)
C11—C10—H10	120.5	C43—C44—H44	120.1
C9—C10—H10	120.5	C45—C44—H44	120.1
C10—C11—C12	120.3 (5)	C46—C45—C44	119.8 (4)
C10—C11—H11	119.8	C46—C45—H45	120.1
C12—C11—H11	119.8	C44—C45—H45	120.1
C7—C12—C11	121.4 (4)	C45—C46—C47	120.8 (4)
C7—C12—H12	119.3	C45—C46—H46	119.6
C11—C12—H12	119.3	C47—C46—H46	119.6
C14—C13—P1	120.0 (3)	C42—C47—C46	120.4 (4)
C14—C13—H13A	107.3	C42—C47—H47	119.8
P1—C13—H13A	107.3	C46—C47—H47	119.8
C14—C13—H13B	107.3	C49—C48—C53	118.2 (4)
P1—C13—H13B	107.3	C49—C48—P4	124.3 (3)
H13A—C13—H13B	106.9	C53—C48—P4	117.3 (3)
C13—C14—C29	112.3 (3)	C48—C49—C50	120.2 (5)
C13—C14—C16	111.2 (3)	C48—C49—H49	119.9
C29—C14—C16	116.4 (3)	C50—C49—H49	119.9
C13—C14—C15	105.5 (3)	C51—C50—C49	121.1 (5)
C29—C14—C15	105.2 (3)	C51—C50—H50	119.5
C16—C14—C15	105.2 (3)	C49—C50—H50	119.5
C14—C15—H15A	109.5	C50—C51—C52	120.1 (5)
C14—C15—H15B	109.5	C50—C51—H51	119.9
H15A—C15—H15B	109.5	C52—C51—H51	119.9
C14—C15—H15C	109.5	C53—C52—C51	119.8 (5)
H15A—C15—H15C	109.5	C53—C52—H52	120.1
H15B—C15—H15C	109.5	C51—C52—H52	120.1
C14—C16—P2	119.4 (3)	C52—C53—C48	120.6 (5)
C14—C16—H16A	107.5	C52—C53—H53	119.7
P2—C16—H16A	107.5	C48—C53—H53	119.7
C14—C16—H16B	107.5	C59—C54—C55	118.6 (4)
P2—C16—H16B	107.5	C59—C54—P4	118.8 (3)
H16A—C16—H16B	107.0	C55—C54—P4	122.6 (3)
C22—C17—C18	118.8 (4)	C54—C55—C56	119.9 (5)
C22—C17—P2	118.9 (3)	C54—C55—H55	120.0
C18—C17—P2	122.3 (3)	C56—C55—H55	120.0
C19—C18—C17	120.0 (4)	C57—C56—C55	120.6 (5)
C19—C18—H18	120.0	C57—C56—H56	119.7
C17—C18—H18	120.0	C55—C56—H56	119.7
C18—C19—C20	121.0 (4)	C56—C57—C58	119.9 (5)
C18—C19—H19	119.5	C56—C57—H57	120.0
C20—C19—H19	119.5	C58—C57—H57	120.0
C21—C20—C19	120.0 (4)	C57—C58—C59	119.2 (5)
C21—C20—H20	120.0	C57—C58—H58	120.4
C19—C20—H20	120.0	C59—C58—H58	120.4
C20—C21—C22	119.5 (4)	C54—C59—C58	121.8 (4)
C20—C21—H21	120.3	C54—C59—H59	119.1
C22—C21—H21	120.3	C58—C59—H59	119.1
C17—C22—C21	120.6 (4)	F2—B1—F4	121.8 (14)
C17—C22—H22	119.7	F2—B1—F3	111.5 (12)
C21—C22—H22	119.7	F4—B1—F3	105.9 (12)
C28—C23—C24	118.6 (4)	F2—B1—F1	110.9 (17)
C28—C23—P2	122.6 (3)	F4—B1—F1	107.4 (13)
C24—C23—P2	118.7 (3)	F3—B1—F1	96.3 (15)
C23—C24—C25	120.6 (4)	F4'—B1—F1'	115.8 (14)
C23—C24—H24	119.7	F4'—B1—F3'	114.9 (12)
C25—C24—H24	119.7	F1'—B1—F3'	118.7 (13)
C26—C25—C24	119.0 (5)	F4'—B1—F2'	104.0 (12)
C26—C25—H25	120.5	F1'—B1—F2'	99.5 (15)
C24—C25—H25	120.5	F3'—B1—F2'	99.5 (11)
			
P4—Cu1—P1—C7	−64.02 (14)	C17—P2—C23—C28	−131.1 (3)
P3—Cu1—P1—C7	68.51 (13)	C16—P2—C23—C28	−23.2 (4)
P2—Cu1—P1—C7	160.59 (13)	Cu1—P2—C23—C28	96.4 (3)
P4—Cu1—P1—C1	64.53 (17)	C17—P2—C23—C24	52.1 (3)
P3—Cu1—P1—C1	−162.94 (17)	C16—P2—C23—C24	160.0 (3)
P2—Cu1—P1—C1	−70.87 (17)	Cu1—P2—C23—C24	−80.4 (3)
P4—Cu1—P1—C13	−178.14 (15)	C28—C23—C24—C25	−2.1 (6)
P3—Cu1—P1—C13	−45.61 (15)	P2—C23—C24—C25	174.8 (3)
P2—Cu1—P1—C13	46.47 (15)	C23—C24—C25—C26	2.3 (6)
P4—Cu1—P2—C17	−52.27 (14)	C24—C25—C26—C27	−0.6 (7)
P3—Cu1—P2—C17	175.71 (13)	C25—C26—C27—C28	−1.2 (8)
P1—Cu1—P2—C17	78.91 (13)	C24—C23—C28—C27	0.3 (6)
P4—Cu1—P2—C23	73.46 (16)	P2—C23—C28—C27	−176.5 (3)
P3—Cu1—P2—C23	−58.56 (16)	C26—C27—C28—C23	1.4 (7)
P1—Cu1—P2—C23	−155.35 (16)	C13—C14—C29—P3	−67.7 (4)
P4—Cu1—P2—C16	−168.27 (14)	C16—C14—C29—P3	62.1 (4)
P3—Cu1—P2—C16	59.71 (15)	C15—C14—C29—P3	178.1 (3)
P1—Cu1—P2—C16	−37.08 (15)	C30—P3—C29—C14	−120.1 (3)
P4—Cu1—P3—C30	−71.03 (14)	C36—P3—C29—C14	134.6 (3)
P2—Cu1—P3—C30	62.05 (14)	Cu1—P3—C29—C14	1.7 (3)
P1—Cu1—P3—C30	159.89 (14)	C36—P3—C30—C31	68.0 (4)
P4—Cu1—P3—C36	55.16 (17)	C29—P3—C30—C31	−39.5 (4)
P2—Cu1—P3—C36	−171.75 (16)	Cu1—P3—C30—C31	−154.5 (3)
P1—Cu1—P3—C36	−73.91 (16)	C36—P3—C30—C35	−110.8 (3)
P4—Cu1—P3—C29	175.10 (14)	C29—P3—C30—C35	141.7 (3)
P2—Cu1—P3—C29	−51.81 (14)	Cu1—P3—C30—C35	26.7 (3)
P1—Cu1—P3—C29	46.02 (14)	C35—C30—C31—C32	−2.0 (6)
P3—Cu1—P4—C42	−172.36 (14)	P3—C30—C31—C32	179.2 (3)
P2—Cu1—P4—C42	69.71 (14)	C30—C31—C32—C33	1.0 (7)
P1—Cu1—P4—C42	−52.92 (14)	C31—C32—C33—C34	0.4 (7)
P3—Cu1—P4—C48	−55.23 (15)	C32—C33—C34—C35	−0.8 (7)
P2—Cu1—P4—C48	−173.16 (14)	C33—C34—C35—C30	−0.2 (6)
P1—Cu1—P4—C48	64.21 (15)	C31—C30—C35—C34	1.6 (6)
P3—Cu1—P4—C54	65.96 (15)	P3—C30—C35—C34	−179.5 (3)
P2—Cu1—P4—C54	−51.97 (15)	C30—P3—C36—C41	−163.0 (3)
P1—Cu1—P4—C54	−174.60 (14)	C29—P3—C36—C41	−55.8 (4)
C7—P1—C1—C2	−160.4 (4)	Cu1—P3—C36—C41	64.9 (4)
C13—P1—C1—C2	−54.3 (4)	C30—P3—C36—C37	23.6 (4)
Cu1—P1—C1—C2	65.0 (4)	C29—P3—C36—C37	130.9 (3)
C7—P1—C1—C6	20.1 (4)	Cu1—P3—C36—C37	−108.5 (3)
C13—P1—C1—C6	126.2 (3)	C41—C36—C37—C38	1.0 (6)
Cu1—P1—C1—C6	−114.5 (3)	P3—C36—C37—C38	174.6 (4)
C6—C1—C2—C3	0.5 (7)	C36—C37—C38—C39	−1.0 (8)
P1—C1—C2—C3	−179.0 (4)	C37—C38—C39—C40	0.1 (8)
C1—C2—C3—C4	0.3 (9)	C38—C39—C40—C41	0.8 (7)
C2—C3—C4—C5	−1.0 (10)	C37—C36—C41—C40	−0.1 (6)
C3—C4—C5—C6	0.8 (9)	P3—C36—C41—C40	−173.8 (3)
C4—C5—C6—C1	0.0 (8)	C39—C40—C41—C36	−0.8 (7)
C2—C1—C6—C5	−0.7 (7)	C48—P4—C42—C47	105.0 (3)
P1—C1—C6—C5	178.9 (4)	C54—P4—C42—C47	−2.6 (4)
C1—P1—C7—C12	−114.6 (3)	Cu1—P4—C42—C47	−132.8 (3)
C13—P1—C7—C12	140.2 (3)	C48—P4—C42—C43	−80.1 (3)
Cu1—P1—C7—C12	24.3 (3)	C54—P4—C42—C43	172.3 (3)
C1—P1—C7—C8	64.9 (3)	Cu1—P4—C42—C43	42.2 (4)
C13—P1—C7—C8	−40.4 (3)	C47—C42—C43—C44	0.7 (7)
Cu1—P1—C7—C8	−156.2 (3)	P4—C42—C43—C44	−174.5 (4)
C12—C7—C8—C9	−2.0 (6)	C42—C43—C44—C45	0.1 (8)
P1—C7—C8—C9	178.5 (3)	C43—C44—C45—C46	−1.4 (8)
C7—C8—C9—C10	2.3 (7)	C44—C45—C46—C47	1.9 (8)
C8—C9—C10—C11	−1.8 (7)	C43—C42—C47—C46	−0.2 (6)
C9—C10—C11—C12	1.1 (7)	P4—C42—C47—C46	174.7 (3)
C8—C7—C12—C11	1.3 (6)	C45—C46—C47—C42	−1.1 (7)
P1—C7—C12—C11	−179.2 (3)	C42—P4—C48—C49	0.4 (4)
C10—C11—C12—C7	−0.9 (6)	C54—P4—C48—C49	108.2 (3)
C7—P1—C13—C14	−127.0 (3)	Cu1—P4—C48—C49	−121.8 (3)
C1—P1—C13—C14	128.0 (3)	C42—P4—C48—C53	176.6 (3)
Cu1—P1—C13—C14	−3.7 (3)	C54—P4—C48—C53	−75.6 (3)
P1—C13—C14—C29	68.2 (4)	Cu1—P4—C48—C53	54.3 (3)
P1—C13—C14—C16	−64.2 (4)	C53—C48—C49—C50	1.1 (6)
P1—C13—C14—C15	−177.7 (3)	P4—C48—C49—C50	177.2 (3)
C13—C14—C16—P2	78.9 (4)	C48—C49—C50—C51	−0.7 (7)
C29—C14—C16—P2	−51.5 (4)	C49—C50—C51—C52	0.2 (8)
C15—C14—C16—P2	−167.5 (3)	C50—C51—C52—C53	−0.1 (8)
C17—P2—C16—C14	−141.1 (3)	C51—C52—C53—C48	0.4 (7)
C23—P2—C16—C14	113.8 (3)	C49—C48—C53—C52	−0.9 (6)
Cu1—P2—C16—C14	−17.1 (3)	P4—C48—C53—C52	−177.3 (4)
C23—P2—C17—C22	−125.5 (3)	C42—P4—C54—C59	−90.2 (3)
C16—P2—C17—C22	127.6 (3)	C48—P4—C54—C59	161.8 (3)
Cu1—P2—C17—C22	10.9 (4)	Cu1—P4—C54—C59	35.9 (4)
C23—P2—C17—C18	54.2 (3)	C42—P4—C54—C55	88.1 (4)
C16—P2—C17—C18	−52.7 (4)	C48—P4—C54—C55	−19.9 (4)
Cu1—P2—C17—C18	−169.4 (3)	Cu1—P4—C54—C55	−145.7 (3)
C22—C17—C18—C19	2.2 (6)	C59—C54—C55—C56	1.2 (6)
P2—C17—C18—C19	−177.5 (3)	P4—C54—C55—C56	−177.1 (4)
C17—C18—C19—C20	−1.3 (7)	C54—C55—C56—C57	−1.2 (7)
C18—C19—C20—C21	−0.5 (7)	C55—C56—C57—C58	0.3 (8)
C19—C20—C21—C22	1.5 (6)	C56—C57—C58—C59	0.6 (8)
C18—C17—C22—C21	−1.3 (6)	C55—C54—C59—C58	−0.4 (7)
P2—C17—C22—C21	178.4 (3)	P4—C54—C59—C58	178.1 (4)
C20—C21—C22—C17	−0.6 (6)	C57—C58—C59—C54	−0.6 (7)

### Hydrogen-bond geometry (Å, °)

**Table:**

*D*—H···*A*	*D*—H	H···*A*	*D*···*A*	*D*—H···*A*
C10—H10···F4^i^	0.93	2.35	3.229 (12)	158

Symmetry codes: (i) −*x*+1, −*y*+1, −*z*+1.
